# In Vitro Anti-Glioblastoma Activity of *Echinocereus engelmannii-* and *Echinocereus pectinatus*-Associated Bacterial Endophyte Extracts

**DOI:** 10.3390/life15040519

**Published:** 2025-03-21

**Authors:** Ana L. Delgado-Miranda, Ricardo Gomez-Flores, Nancy E. Rodríguez-Garza, Orquídea Pérez-González, Patricia Tamez-Guerra, Diana Caballero-Hernández, Diana L. Clark-Pérez, Ramiro Quintanilla-Licea, Andrés García, César I. Romo-Sáenz

**Affiliations:** 1 Departamento de Microbiología e Inmunología, Facultad de Ciencias Biológicas, Universidad Autónoma de Nuevo León, San Nicolás de los Garza 66455, Nuevo León, Mexico; laura.delgadom@uanl.edu.mx (A.L.D.-M.); nancy.rodriguezg@usal.es (N.E.R.-G.); operezg@uanl.edu.mx (O.P.-G.); patricia.tamezgr@uanl.edu.mx (P.T.-G.); diana.caballerohr@uanl.edu.mx (D.C.-H.);; 2 Grupo de Enfermedades Infecciosas y Tropicales (e-INTRO), Facultad de Farmacia, Instituto de Investigación Biomédica de Salamanca-Centro de Investigación de Enfermedades Tropicales de la Universidad de Salamanca (IBSAL-CIETUS), Universidad de Salamanca, 37007 Salamanca, Spain; 3 Departamento de Química, Facultad de Ciencias Biológicas, Universidad Autónoma de Nuevo León, San Nicolás de los Garza 66455, Nuevo León, Mexico; ramiro.quintanillalc@uanl.edu.mx; 4 Laboratorio de Biotecnología Ambiental, Centro de Investigación en Biotecnología, Universidad Autónoma del Estado de Morelos, Cuernavaca 62209, Morelos, Mexico; 5 Facultad de Medicina y Ciencias Biomédicas, Universidad Autónoma de Chihuahua, Chihuahua 31109, Chihuahua, Mexico

**Keywords:** *Stenotrophomonas maltophilia*, *Echinocereus engelmannii*, *Echinocereus pectinatus*, antitumor activity, cytotoxic activity

## Abstract

Glioblastoma is the most common and aggressive brain tumor in adults. However, due to the limitations of conventional treatments, as well as their side effects, there is a need to develop more effective and less harmful therapy strategies. There is evidence that plants endemic to northern Mexico possess biological activities that positively impact human health, particularly against cancer. *Echinocereus engelmannii* and *Echinocereus pectinatus* are cacti from the north of Mexico that produce bioactive compounds with antitumor activity. We obtained methanol extracts from previously isolated and fermented microorganisms associated with these cacti. Cell lines of extracts with cytotoxicity against glioblastoma cells U87, neuroblastoma cells SH-S5Y5, and Schwann neuronal cells (healthy control) were evaluated, using a colorimetric 3-(4,5-dimethylthiazol-2-yl)-2,5-diphenyltetrazole bromide (MTT) reduction technique. The selective cytotoxicity extracts were analyzed using liquid chromatography tandem mass spectrometry (LC/MS^2^). We isolated 19 endophytic and soil-associated microorganisms from both cacti. Two of them were selected for their high percentages of tumor growth inhibition. The microorganism ES4 possessed the best activity with an IC_50_ of 17.31 ± 1.70 µg/mL and a selectivity index of 3.11. We identified the bacterium *Stenotrophomonas maltophilia* by matrix-assisted laser desorption/ionization time-of-flight mass spectrometry (MALDI-TOF MS) from the most active microorganisms against tumor growth. LC/MS^2^ characterized the HS4 extract, and the most abundant group (50.0%) identified included carboxylic acids and derivatives, particularly bisgerayafolin A, Cyclo (Pro-Leu), maculosin, and tryptophan. In conclusion *S. maltophilia* extract inhibit the growth of glioma cells, showing greater sensitivity in the U87 cell line.

## 1. Introduction

Cancer is the leading cause of death worldwide, representing the 10th most common cause of death, with gliomas being the most prevalent type of malignant brain tumors. The first symptoms in adults are not specific, involving headaches, seizures, and vision problems, among others, making timely diagnosis difficult [1]. Although multiple treatments have been developed against this type of disease, the death rate remains at 9,743,832 deaths per year and is estimated to increase to 16,884,723 by 2045 [2]. This is due to the resistance to currently existing drugs and the high rate of mutations of diverse types of cancer. In Mexico in 2018, central nervous system (CNS) tumors were the 17th in cancer incidence with 3451 cases and the 13th in cancer deaths [3].

Glioblastoma is a tumor that directly affects the CNS and is considered the most common and aggressive brain tumor in adults [4]. Globally, 248,500 deaths from this type of cancer were estimated in 2022, and by 2045, a 54.1% increase in the mortality rate is expected [2]. Current treatment consists of complete surgical removal of the tumor followed by a combination of chemotherapy and radiotherapy [5]. However, the difficulty of a timely diagnosis, as well as the resistance to the most commonly used chemotherapeutics such as temozolomide [6] are related to the development of more aggressive cancers, directly impacting patient survival or increasing tumor regression in most patients. Due to the different mechanisms of resistance to treatments and the high rate of mutations in glioblastoma, the search for new cancer treatments is a constant challenge [7].

Mexican plants have been used as traditional remedies for different health conditions and have shown to possess anticancer potential and less toxicity compared with conventional therapies [8,9]. In recent years, various agents have been isolated and used in the treatment of distinct types of cancer, including vinblastine, vincristine, podophyllotoxin, paclitaxel (Taxol), and camptothecin, which have been extensively studied both chemically and clinically [10].

Cacti are promising producers of anticancer molecules due to their potential to grow under extreme climate and water scarcity environments. Consequently, they create natural defenses such as phytochemicals, including alkaloids, flavonoids, terpenes, and tannins, potentially generated by endophytic microorganisms, which possess relevant bioactivities against cancer [8,9].

Within the Cactaceae family, there is evidence of several highly studied species, such as *Opuntia* spp., which, besides being a food of great nutritional importance, possesses anticancer, antioxidant, anti-inflammatory, antiviral, and antidiabetic potential. These bioactivities have been observed in both stem and fruit extracts of these species, which demonstrated good effectiveness in different in vitro and in vivo models [11].

An important antioxidant activity has been observed in species of *Echinocereus*, as well as a high content of phenolic compounds, such as quercetin and isorhamnetin, along with their derivatives, protocatechuic acid and apigenin [9]. It has been shown that quercetin induces apoptosis in liver cancer tumor cells (HepG2) when different flavonoid concentrations are added. Higher doses of these substances were shown to induce cell death through increased activation of caspases 3 and 9, as well as inhibition of the AKT kinase protein and the phosphorylation of the ERK kinase protein. In addition, they produced an increase in the pro-apoptotic factors Bcl-2 and a decrease in the anti-apoptotic Bcl-XL [12].

Endophytic or rhizosphere microorganisms from plants have gained relevance as potential sources for identifying new compounds with antitumor activity. Their rapid growth, culture conditions, high cell density, easy genetic manipulation, and the possibility of scaling up the production of compounds to an industrial level make them potential candidates for obtaining anticancer drugs with significant efficiency [13]. There are several reports on the biological activity of endophytic microorganisms, particularly those associated with *Lophocereus marginatus*, such as *Aspergillus versicolor* and *Metarhizium anisopliae*, as well as endophytic microorganisms from *Ibervillea sonorae*, including *Micromonospora echinospora* and *Bacillus subtilis*, have demonstrated anticancer activity in both in vivo and in vitro murine lymphoma models [13,14,15].

The present study aimed to determine the in vitro antitumor potential of endophytic microorganism extracts from the cacti *Echinocereus engelmanni* (Parry ex Engelm.) Lem. and *E. pectinatus* (Scheidw.) Engelm. against glioblastoma.

## 2. Materials and Methods

### 2.1. Plant Material

The mature size cacti *Echinocereus engelmanni* and *E. pectinatus* used in this study were obtained from the greenhouse “Vivero Ortiz” in Chihuahua, Chihuahua, México on August 21, 2021. Taxonomic identification of the plant was performed at the herbarium of the Facultad de Ciencias Biológicas of the Universidad Autónoma de Nuevo León (FCB-UANL), with a voucher number 25608 for *E. pectinatus* and 25607 for *E. engelmanii*.

Taxonomic validation of plant names and families was performed using the International Plant Names Index (https://www.ipni.org/; accessed on 2 March 2025).

### 2.2. Isolation of Endophytic Microorganisms from E. engelmannii and E. pectinatus

Endophytic microorganisms from the cactus body stem and soil-associated root were isolated through the following steps. The root was washed with soap and water and internal cuts of 1 cm^2^ were made, followed by 1-min washes in 1% NaClO and sterile distilled water, a 1-min treatment with 70% ethanol, two consecutive 1-min rinses with sterile distilled water, and a final wash with PBS solution (negative control), all under sterile conditions in a biosafety level 2 laminar-flow hood. Disinfected root pieces were incubated on potato dextrose agar plates (PDA; Titan biotech LTD, Bhiwadi, India) for two weeks at 28 °C ± 2 °C. The plant tissue was then macerated in 9 mL of PBS, using a sterile mortar, and 100 μL of the macerate were plated on PDA and incubated for two weeks at 28 °C ± 2 °C. Isolated colonies (colonies began to form after 10 days) were stored in PDB with 30% glycerol at −70 °C, until use [15].

We isolated 19 endophytic (E) and soil-associated (S) microorganisms, including three soil-associated microorganisms (HS1, HS4, and HS5) and two endophytes (HE1 and HE2) from *Echinocereus engelmannii,* and 10 soil-associated microorganisms (ES4 to ES13) and four endophytes (EE1 to EE4) from *E. pectinatus*.

### 2.3. Methanol Extracts Preparation and Fermentation of Endophytic Microorganisms from E. engelmannii and E. pectinatus

Nineteen microorganisms were isolated from the cacti and fermented, after which methanol extracts were obtained. For this, pieces of two weeks PDA-cultured endophytic microorganisms agar (2 cm^2^ pieces) were obtained and placed in flasks with 150 mL of potato dextrose broth (PDB; BD, Becton, Dickinson and Company, Sparks, MD, USA), which were statically fermented at 28 °C ± 2 °C for three weeks. The biomass obtained was then filtered through filter paper, and methanol was added and kept under agitation for 48 h at 28 °C ± 2 °C. Next, extracts were filtered and concentrated by rotary evaporation with a vacuum rotary evaporator (Buchi R-3000; Brinkman Instruments, Inc., Westbury, NY, USA). The residual solvent was removed with a SpeedVac concentrator (Thermo Fisher Scientific, San Jose, CA, USA) at 35 °C [8,9]. The extraction yield was calculated using the following formula: % Yield = (weight of dry extracts/weight of vegetal material) × 100. Next, 100 mg of each extract was dissolved in one milliliter of dimethyl sulfoxide (DMSO; Sigma-Aldrich, St. Louis, MO, USA), sterilized by filtration, using 0.22 μm pore size membrane filters (Corning Incorporated, Corning, NY, USA), and stored at −20 °C until use. The final concentration of DMSO used in cell cultures was less than 1% (*v*/*v*), which did not affect cell viability [13].

### 2.4. Cell Lines and Culture Conditions

In this study, we used the glioblastoma cells U87 (ATCC HTB-14), neuroblastoma cells SH-S5Y5 (ATCC CRL-2266), and Schwann neuronal cells (ATCC CRL-3392; healthy control). They were maintained in Dulbecco’s modified Eagle’s medium (DMEM; Sigma-Aldrich), supplemented with 10% fetal bovine serum (FBS; Grand Island, NY, USA) that was previously heat-inactivated and 1% antibiotic antimycotic solution (Sigma-Aldrich, St. Louis, MO, USA) at 37 °C and 5% CO_2_ in a humidified atmosphere.

### 2.5. Effect of E. engelmannii and E. pectinatus-Associated Endophytes on Tumor Cell Growth

We incubated 190 µL of U87, SH-S5Y5, and Schwann cell suspensions (1 × 10^4^ cells/well) in DMEM medium in 96-well flat-bottomed microplates (Corning Incorporated) for 24 h, after which we added the methanol extracts at concentrations of 31.25 µg/mL to 250 µg/mL for an initial selection. We used paclitaxel at 0.1 µM (0.0854 µg/mL) as a positive control and culture medium without treatment as a negative control. Next, culture plates were incubated for 48 h at 37 °C and 5% CO_2_ in a humid atmosphere. Cell viability was then determined by the colorimetric 3-[4,5-dimethylthiazol-2-yl]-2,5-diphenyltetrazoliumbromide (MTT; Affymetrix, Cleveland, OH, USA) method by adding 15 µL of MTT (0.5 mg/mL final concentration) to each well and incubating for three hours at 37 °C and 5% CO_2_. After this, plates were decanted, and 100 µL of DMSO were added to dissolve formazan crystals. Optical densities (OD) were then measured at 570 nm in a microplate reader (MULTISKAN GO; Thermo Fisher Scientific, Waltham, MA, USA). The percentage of growth inhibition was determined by the following formula: % Growth inhibition = 100 − (OD_570_ treated cells/OD_570_ untreated cells) × 100.

Based on the results obtained, we selected the two microorganism extracts with the highest growth inhibition percentage (HS4 and ES4). Concentrations ranging from 1 µg/mL to 250 µg/mL were evaluated. We then used the growth inhibition concentration-response curve to calculate the half-maximal inhibitory concentration (IC_50_) and the selectivity index (SI) of each extract using the following formula: SI = IC_50_ normal cells (Schwann cells)/IC_50_ tumor cells (U87 and SH-S5Y5 cells).

### 2.6. Antioxidant Activity

We also evaluated the antioxidant activity of HS4 and ES4. For this, we incubated 100 μL of the extracts in 96-well flat-bottomed plates at concentrations ranging from 3.9 µg/mL to 250 µg/mL and 100 μL of a 0.1 mM methanol solution of 2,2-diphenyl-1-picrylhydrazyl (DPPH) at 28 °C ± 2 °C for 30 min in darkness, using DMSO as the negative control and 10 μg/mL to 100 μg/mL ascorbic acid as the positive control. ODs were then read at 517 nm in a Genesys 20 spectrophotometer (Thermo-Fisher Scientific, Waltham, MA, USA). DPPH reduction percentage was calculated using the following formula: % DPPH reduction = [(OD_517_ negative control − OD_517_ treatment)/OD_517_ negative control] × 100 [15].

### 2.7. Hemolytic Activity Test

The in vitro hemolysis test is used to evaluate the toxicity of a variety of products in the pharmaceutical industry to be applied in the clinic. We then determined the hemolytic activity of HS4 and ES4. For this test, we obtained 20 mL of blood from a healthy volunteer in BD Vacutainer EDTA tubes (Becton Dickinson, Franklin Lakes, NJ, USA), after which blood was centrifuged to separate erythrocytes from plasma at 1500 rpm (15 min at 25 °C) and washed three times with 10 mL of PBS (pH 7.4). Erythrocytes were then suspended at 5% *v*/*v* in PBS for the hemolytic activity test, for which this suspension was mixed with the extracts dissolved in PBS at concentrations ranging from 200 μg/mL to 1000 μg/mL in 2 mL Eppendorf (Eppendorf^®^ AG, Hamburg, Germany) microcentrifuge tubes for 30 min at 37 °C in darkness, using distilled water as a positive control and PBS as a negative control. Samples were incubated for 30 min at 37 °C, after which they were centrifuged for five minutes at 13,000 rpm and 4 °C. Next, 200 µL of supernatant fluids were obtained from each tube and transferred to 96-well flat-bottomed plates to measure the OD of the released hemoglobin at 540 nm in a microplate reader [16]. The percentage of hemolysis was calculated using the following formula: % Hemolysis = [(OD_540_ treatment − OD_540_ negative control)/(OD_540_ positive control − OD_540_ negative control)] × 100.

### 2.8. Identification of Selected Strains

HS4 and ES4 strains were sent to the Infectious Diseases Laboratory of the University Hospital “Dr. José Eleuterio González”, Autonomous University of Nuevo Leon, Mexico, where they were identified via matrix-assisted laser desorption/ionization time-of-flight mass spectrometry (MALDI-TOF MS) (Microflex LT System, BrukerDaltonics, Bremen, Germany), using the MALDI Biotyper 3.0 software.

### 2.9. Characterization of Methanol Extracts of Selected Microorganisms

Extract characterization was performed in the Metabolomics and Proteomics Specialized Laboratory (MetPro) of Ensenada, Baja California, Mexico, using liquid chromatography-tandem mass spectrometry (LC/MS^2^).

We chose the HS4 extract as it exhibited the highest activity in the U87 cell line. For the analysis, the HS4 methanol extract was suspended in a water-acetonitrile mixture (80:20) and injected (2 μL or 600 ng) into an Agilent 1260 Infinity LC (Agilent Technologies, Inc., Santa Clara, CA, USA). The LC/MS^2^ analysis followed the protocol previously described.

For MS data acquisition, positive mode nanospray ionization was used with the following conditions: capillary voltage (1850 V), gas temperature (350 °C), drying gas flow (5 L/min), skimmer voltage (65 V), octapole RF (750 V), fragmentor voltage (175 V), and spectra acquisition rate (4 spectra/s) over a mass range of 110–2000 *m*/*z*. For MS^2^ data, a narrow isolation window (1.3 *m*/*z*), spectra acquisition rate (3 spectra/s), and a maximum of 5 precursors per cycle in the 50–2000 *m*/*z* range were applied. Active exclusion was enabled for two spectra with a release time of 0.25 min. Collision energy was ramped with slope and offset values of 6 and 4, respectively.

The LC/MS^2^ datasets were analyzed using open-access software and online platforms. The Global Natural Products Social Molecular Networking web platform (GNPS, https://gnps.ucsd.edu, accessed on 5 March 2023) was used for automated structural analysis. Additionally, the CSI:FingerID package from SIRIUS software version 4.9.12 (https://bio.informatik.uni-jena.de/software/sirius/, accessed on 5 March 2023) was employed as a complementary tool. The chemical classes of the identified metabolites were automatically determined using the Classyfire web-based application (http://classyfire.wishartlab.com, accessed on 10 March 2023) [16].

### 2.10. Statistical Analysis

Statistical analyses were performed using the Graph Pad Prism 8 program (GraphPad Software Inc., San Diego, CA, USA). A nonlinear regression analysis was used to calculate the half maximal inhibitory concentration (IC_50_) values. All data represent means ± standard deviations (SD) of triplicate determinations from three independent experiments.

## 3. Results

### 3.1. Isolation of Microorganisms and Extraction Yields

We isolated nineteen endophytic (E) and soil-associated (S) microorganisms. Endophytic microorganisms were isolated from the body stem of the cactus and soil-associated with roots. These microorganisms include three soil-associated microorganisms (HS1, HS4, and HS5) and two endophytes (HE1 and HE2) from *E. engelmannii*, and ten soil-associated microorganisms (ES4 to ES13) and four endophytes (EE1 to EE4) from *E. pectinatus*, whose yields are shown in Table 1.

### 3.2. Effect of Extracts on Tumor Cell Growth

**Figure 1:**
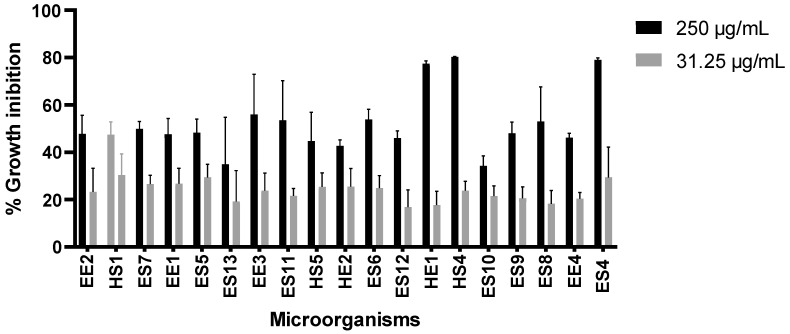
Effect of microorganism extracts on U87 cell growth. Paclitaxel was used as a positive control at a concentration of 0.1 µM (0.0854 µg/mL), causing 80% growth inhibition, and cells without treatment as a negative control.

**Figure 2:**
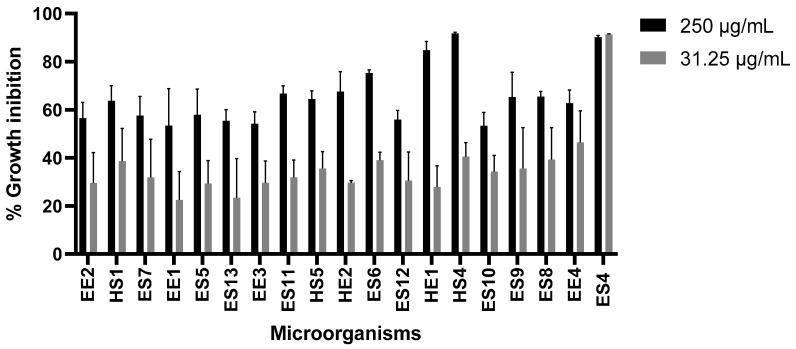
Effect of microorganism extracts on SH-SY5Y cell growth. Paclitaxel was used as a positive control at a concentration of 0.1 µM (0.0854 µg/mL), causing 65% growth inhibition, and cells without treatment as a negative control.

We initially evaluated 31.25 µg/mL and 250 µg/mL of microorganism extracts to select those with the highest activity against U87 (Figure 1) and SH-S5Y5 (Figure 2) cells. We found that ES4 and HS4 possessed the highest growth inhibition activity.

As HS4 and ES4 microorganisms extracts showed the highest inhibition activity concentration-related growth response curves were produced with these microorganisms extracts to obtain the IC_50_ and SI. The IC_50_ and SI results of the extracts from the microorganisms ES4 and HS4 against the U87 and SH-S5Y5 cell lines are shown in Table 2 and Table 3, respectively. Results for ES4 showed a higher antitumor potential in the SH-S5Y5 cell line with an IC_50_ of 17.31 µg/mL, compared to the U87 cell line with an IC_50_ of 45.11 µg/mL. The microorganism extract HS4 showed a higher antitumor potential in the U87 cell line with an IC_50_ of 87.66 µg/mL compared to the SH-S5Y5 cell line with an IC_50_ of 117.90 µg/mL.

### 3.3. Antioxidant and Hemolytic Activities

Extracts of the two microorganisms extracts with the highest antitumor activity were selected to evaluate their antioxidant and hemolytic activities. They showed IC_50_ > 250 µg/mL, thus indicating marginal antioxidant or hemolytic activities (Table 4).

### 3.4. Identification of Selected Microorganisms

The selected microorganisms were identified using mass spectrometry (MALDI-TOF) (Table 5). Both microorganisms were identified as *Stenotrophomonas maltophilia* with a different score for each strain: 1.93 for HS4 and 1.54 for ES4.

### 3.5. Characterization of Methanol Extracts

**Figure 3:**
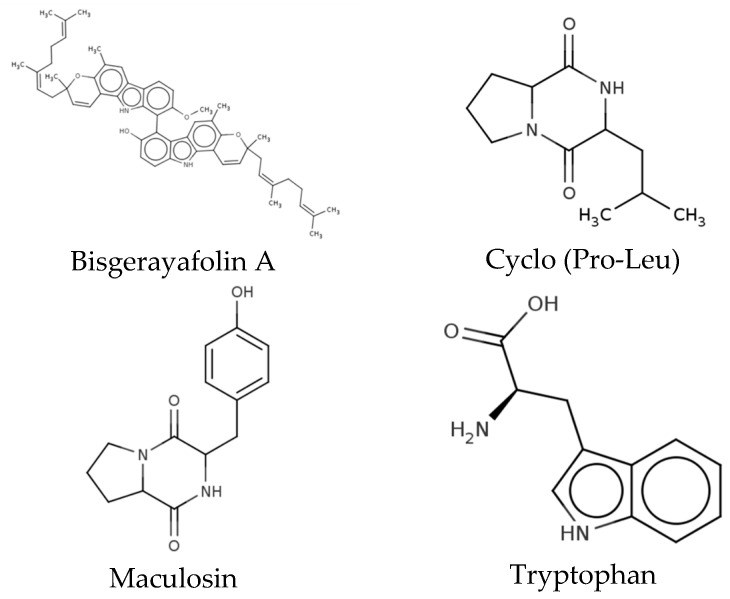
Metabolites present in the HS4 methanol extract with biological activity reported.

The extract produced by the microorganism HS4 was characterized by LC/MS^2^. Three main groups of bioactive compounds were found, the most abundant being carboxylic acids and derivatives (50.0%), followed by glycerophospholipids (33.33%) and indoles and derivatives (16.67%). Specifically, 12 metabolites were identified as cyclo (Pro-Val), Lyso PE (16:0/0:0), N-fructosyl isoleucine, PE (15:0/16:0), PE (16:0/14:0), PE (16:0/16:1), Fen-Pro, pyroglutamylproline, bisgerayafolin A, cyclo (Pro-Leu), maculosin, and tryptophan (Table 6). Structure of compound with biological activity reported are shown in Figure 3.

## 4. Discussion

Glioblastoma is the most common brain tumor in adults [17] surgical removal, followed by chemotherapy with temozolomide (C_6_H_6_N_6_O_2_), in combination with radiotherapy [18]. Temozolomide is the chemotherapeutic agent of choice against glioblastoma. It is a dacarbazine derivative that presents alkylating activity. It shows relatively good therapeutic efficacy but presents several side effects [19]. Despite this treatment, most tumors are incurable and highly recurrent because conventional therapies are not usually effective against tumor cells and damage healthy cells [19]. This has led to the search for new alternative approaches, such as the use of natural products. In this context, there are natural compounds obtained from cacti such as opuntiol, macdougallin, and peniocerol, which have shown significant antitumor potential [20,21]. However, in recent years it has been shown that in certain cases, the antitumor activity of plants is provided by endophytic microorganisms [22]. Therefore, in the present study we investigated the antitumor potential of crude methanol extracts obtained from endophytic microorganisms of *Echinocereus engelmannii* and *E. pectinatus*.

Nineteen microorganisms with different morphological characteristics were isolated. We obtained up to 6.30% crude methanol extract yields from these microorganisms, which were higher than those obtained from our previous study using endophytes isolated from the cactus *Ibervillea sonorae,* with up to 3.5% [15]. The antitumor effect of these microorganisms was subsequently evaluated, showing higher activity against the SH-S5Y5 cell line as compared with that against the U87 cell line. This has been previously reported, where the bacoside compound extracted from the plant *Bacopa monniera* caused 60% SH-S5Y5 growth inhibition [22], and may be due to the presence of new mutations and oncogenic expressions for drug resistance in different models of human glioblastoma and that resistance to the drug temozolomide has also been observed in the U87 cell line, resulting in treatment failure [23].

Of the 19 microorganism extracts evaluated, HE1, HS4, and ES4 presented the highest antitumor activity, with a growth inhibition >80% in both glioblastoma cell lines. These results highlight the importance of evaluating the antitumor potential of endophytic microorganisms against other cancer cell lines, such as U87, but we have not found evidence of the evaluation of plant-derived endophytic microorganism extracts against glioblastoma. Regarding the HS4 extract, a higher growth inhibition was observed against the cell line SH-S5Y5 (IC_50_ = 87.66 ± 1.54 µg/mL) than that against the U87 cell line (IC_50_ = 117.90 ± 1.99 µg/mL) and an SI of 1.30 for the SH-S5Y5 cell line, as compared with 1.75 for the U87 cell line. Furthermore, the ES4 microorganism extract caused an IC_50_ of 17.31 ± 1.73 µg/mL against the SH-S5Y5 cell line and an IC_50_ of 45.11 ± 1.87 against the U87 cell line, with an SI of 3.11 for the SH-S5Y5 cell line and 1.19 for the U87 cell line. SI > 2 indicating that an extract has pharmacological potential to continue with the fractionation and identify the presence of the compound or compounds responsible for the antitumor activity [24] and a low cellular toxicity to normal cells [25].

In addition, HS4 and ES4 microorganisms extracts showed DPPH values IC_50_ > 250 µg/mL. Extracts with an IC_50_ ≤ 50 µg/mL are considered to have relevant antioxidant activity, thus these extracts do not possess antioxidant potential [13,26]. Regarding the hemolysis analysis, the extracts of both microorganisms showed IC_50_ values > 250 µg/mL, thus they did not have significant hemolysis [27].

HS4 and ES4 microorganisms were identified by MALDI-TOF, determining that they corresponded to the species *Stenotrophomonas maltohpilia*. This species is associated with plants and has been isolated from the rhizosphere or the internal tissues of plants [28]. Moreover, endophytic strains of *S. maltophilia* have been isolated from the roots of many plant species, which possess an extraordinary range of activities, including plant protection and growth promotion, bioremediation, phytoremediation, and the production of biomolecules of significant economic value [29].

Since the HS4 miscoorganism extract presented the highest antitumor activity against U87 cells, it was characterized by LC/MS^2^. The analysis showed a major composition of carboxylic acids and their derivatives. These are commonly used as cancer therapies, as well as to prevent the side effects of radiotherapy [30]. This chemical group has also been described in the endophytic bacteria of rice, demonstrating antifungal activity [31] a high percentage of glycerophospholipids, often used as supplements to restore cellular functions, improve mitochondrial membrane potential, and increase resistance to oxidative stress [32]. Another group identified were indoles and derivatives. It has been shown that several indole-based compounds may have high potential in cancer treatments [33].

In addition, we identified 12 metabolites from HS4 microorganisms extract, among which bisgerayafolin A. It has been reported to have cytotoxic and antioxidant activity, and its presence has been found in plants such as *Murraya koenigii*; however, there is no report on its production by endophytes [34]. Carbazoles, the group to which bisgerayafolin A belongs, possess antimicrobial, anti-inflammatory, antitumor, antihistamine, analgesic, antitumor, and neuroprotective properties [35]. Another important compound identified in the extract was maculosin, which has been used as an antibacterial and antioxidant agent and is also produced by the fungus *Alternaria* [36,37]. Furthermore, this compound has cytotoxic activity against human liver cancer, which is why it is proposed as a compound with a high therapeutic value [38]. Moreover, the dipeptide Cyclo (Pro-Leu) has been shown to have antifungal and antimicrobial activities [39]; tryptophan has been involved in the regulation of immunity, neuronal function, and intestinal homeostasis [40] N-fructosyl isoleucine has been reported to possess antifungal, plant growth regulating, and cell signaling activity, but its antitumor activity has not been investigated [41,42]. The biological activity of the compounds PE (15:0/16:0), PE (16:0/14:0), PE (16:0/16:1), Fen-Pro, and pyroglutamylproline have not yet been elucidated.

However, it is necessary to perform a biotargeted fractionation to isolate and identify the bioactive compounds with antitumoral activity. Additionally, it would be essential to assess the compounds in an in vivo murine model to confirm their antitumoral activity and exclude any potential toxicity.

## 5. Conclusions

These results demonstrate the antitumor effect of methanolic extracts obtained from *S. maltophilia* associated with *E. engelmannii* and *E. pectinatus*, showing significant cytotoxic activity against human glioblastoma cells. Methanolic extracts of *S. maltophilia* are proposed for the study and isolation of new compounds with antitumor activity.

## Figures and Tables

**Table 1:** Methanol extracts yield from microorganisms associated with *E. engelmannii* and *E. pectinatus.*

Microorganism	Yield (%)
ES4	1.17
ES5	0.79
ES6	0.67
ES7	6.30
ES8	0.77
ES9	2.73
ES10	1.46
ES11	0.90
ES12	0.50
ES13	0.56
EE1	0.36
EE2	1.34
EE3	0.79
EE4	2.70
HE1	0.67
HE2	0.57
HS1	0.71
HS4	3.63
HS5	0.56

**Table 2:** Antitumoral activity of HS4 and ES4 extracts against U87 cell line.

Microorganism		IC_50_ (µg/mL)		SI
	Schwan Healthy Cell Line	U87 Tumoral Cell Line		
HS4	152.80 ± 2.20	87.66 ± 1.54		1.75
ES4	53.87 ± 1.73	45.11 ± 1.87	1.19	

Data are mean ± SD of the IC_50_ (µg/mL) for each extract against Schwan and U87 cells at 72 h. SI represents IC_50_ of Schwan cells divided by IC_50_ of U87 cells at 72 h. Paclitaxel was used as a positive control at 0.0854 µg/mL.

**Table 3:** Antitumoral activity of HS4 and ES4 extracts against SH-S5Y5 cell line.

Microorganism	IC_50_ (µg/mL)		SI
	Schwan Healthy Cell Line	SH-S5Y5 Tumoral Cell Line	
HS4	152.80 ± 2.20	117.90 ± 1.99	1.30
ES4	53.87 ± 1.73	17.31 ± 1.73	3.11

Data are mean ± SD of the IC_50_ (µg/mL) for each extract against Schwan and SH-S5Y5 cells at 72 h. SI represents IC_50_ of Schwan cells divided by IC_50_ of SH-S5Y5 cells at 72 h. Paclitaxel was used as a positive control at 0.0854 µg/mL.

**Table 4:** Antioxidant and hemolytic activities of ES4 and HS4 extracts.

Microorganism	IC_50_ (μg/mL)	
	DPPH	Hemolysis
HS4	>250	>250
ES4	>250	>250

**Table 5:** Identification of selected strains by MALDI-TOF.

Microorganism	Strain	Score
HS4	*Stenotrophomonas maltophilia*	1.93
ES4	*Stenotrophomonas maltophilia*	1.54

Score: Numerical scoring for the interpretation of MALDI-TOF results. A score ≥ 1.80 indicates a high confidence level in accurate identification.

**Table 6:** LC/MS^2^ compounds in HS4 extract.

No.	Compound	Experimental Mass	Theoretical Mass	Adduct	Mass Error (ppm)	Formula	RT	Chemical Class
1	N-Fructosyl isoleucine	294.155	294.155	[M + H]^+^	1.18	C_12_H_23_NO_7_	1.3	CAD
2	Pyroglutamylproline	227.103	227.103	[M + H]^+^	1.95	C_10_H_14_N2O_4_	1.7	CAD
3	Tryptophan	205.096	205.097	[M + H]^+^	−5.25	C_11_H_12_N_2_O_2_	3.2	IND
4	Maculosin	261.123	261.123	[M + H]^+^	−1.12	C_14_H_16_N_2_O_3_	3.4	CAD
5	Cyclo(Pro-Val)	197.128	197.128	[M + H]^+^	−1.92	C_10_H_16_N_2_O_2_	3.8	CAD
6	Cyclo(Pro-Leu)	211.144	211.144	[M + H]^+^	−0.13	C_11_H_18_N_2_O_2_	8.8	CAD
7	Phe-Pro	245.129	245.129	[M − H_2_O + H]^+^	−0.01	C_14_H_18_N_2_O_3_	10.9	CAD
8	Lyso PE(16:0/0:0)	454.293	454.293	[M + H]^+^	0.57	C_21_H_44_NO_7_P	26.4	GPL
9	Bisgerayafoline A	815.486	815.478	[M + H]^+^	9.62	C_55_H_62_N_2_O_4_	28.8	IND
10	PE(15:0/16:0)	678.507	678.507	[M + H]^+^	0.36	C_36_H_72_NO_8_P	28.8	GPL
11	PE(16:0/14:0)	664.492	664.491	[M + H]^+^	1.35	C_35_H_70_NO_8_P	29	GPL
12	PE(16:0/16:1)	690.505	690.507	[M + H]^+^	−2.54	C_37_H_72_NO_8_P	29.8	GPL

RT: Retention time in minutes; CAD: Carboxylic acids and derivatives; IND: Indoles and derivatives; GPL: Glycerophospholipids.

## Data Availability

The datasets generated or analyzed during the present study are available from the corresponding authors.
